# The Multifaceted Role of the Lysosomal Protease Cathepsins in Kidney Disease

**DOI:** 10.3389/fcell.2017.00114

**Published:** 2017-12-19

**Authors:** Pasquale Cocchiaro, Valeria De Pasquale, Rossella Della Morte, Simona Tafuri, Luigi Avallone, Anne Pizard, Anna Moles, Luigi Michele Pavone

**Affiliations:** ^1^Department of Molecular Medicine and Medical Biotechnology, University of Naples Federico II, Naples, Italy; ^2^Faculty of Medicine, Institut National de la Santé Et de la Recherche Médicale, “Défaillance Cardiaque Aigüe et Chronique”, Nancy, France; ^3^Université de Lorraine, Nancy, France; ^4^Institut Lorrain du Coeur et des Vaisseaux, Center for Clinical Investigation 1433, Nancy, France; ^5^CHRU de Nancy, Hôpitaux de Brabois, Nancy, France; ^6^Department of Veterinary Medicine and Animal Productions, University of Naples Federico II, Naples, Italy; ^7^Institute of Cellular Medicine, Newcastle University, Newcastle upon Tyne, United Kingdom

**Keywords:** cathepsins, acute kidney injury, chronic kidney disease, lysosomal proteases, signaling pathways

## Abstract

Kidney disease is worldwide the 12th leading cause of death affecting 8–16% of the entire population. Kidney disease encompasses acute (short-lasting episode) and chronic (developing over years) pathologies both leading to renal failure. Since specific treatments for acute or chronic kidney disease are limited, more than 2 million people a year require dialysis or kidney transplantation. Several recent evidences identified lysosomal proteases cathepsins as key players in kidney pathophysiology. Cathepsins, originally found in the lysosomes, exert important functions also in the cytosol and nucleus of cells as well as in the extracellular space, thus participating in a wide range of physiological and pathological processes. Based on their catalytic active site residue, the 15 human cathepsins identified up to now are classified in three different families: serine (cathepsins A and G), aspartate (cathepsins D and E), or cysteine (cathepsins B, C, F, H, K, L, O, S, V, X, and W) proteases. Specifically in the kidney, cathepsins B, D, L and S have been shown to regulate extracellular matrix homeostasis, autophagy, apoptosis, glomerular permeability, endothelial function, and inflammation. Dysregulation of their expression/activity has been associated to the onset and progression of kidney disease. This review summarizes most of the recent findings that highlight the critical role of cathepsins in kidney disease development and progression. A better understanding of the signaling pathways governed by cathepsins in kidney physiopathology may yield novel selective biomarkers or therapeutic targets for developing specific treatments against kidney disease.

## Introduction

**Figure 1:**
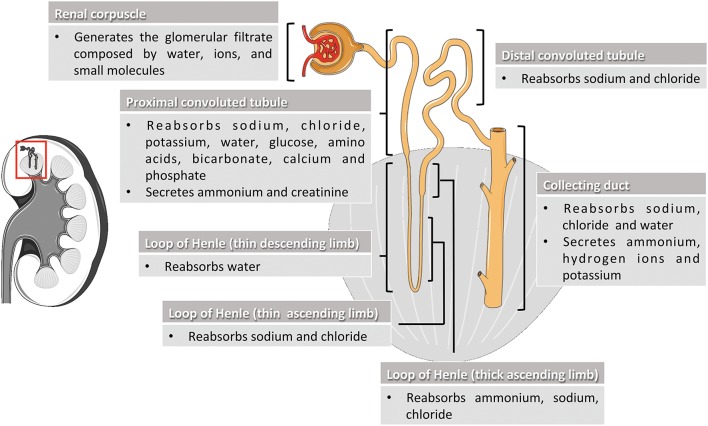
Nephron segments and their main physiological function. The nephron is the functional unit of the kidney and is composed by the renal corpuscle and the renal tubule. In the renal corpuscle, the glomerular filtrate is generated by filtration of water, ions, and small molecules from the bloodstream. The glomerular filtrate is transformed into urine by reabsorption and secretion of different molecules through the different sections of the renal tubule (proximal convoluted tubule, loop of Henle, and distal convoluted tubule) and the collecting duct system. Kidney and nephron image adapted from Smart Servier Medical Art under Creative Commons Attribution 3.0 Unported License.

Kidneys are complex organs whose excretory, biosynthetic and metabolic activities are essential for healthy living. They regulate body fluid balance, blood pressure, waste removal, and red blood cells production (Preuss, 1993; Adamson, 1996). Kidney functions take place through mechanisms of filtration, reabsorption and secretion occurring in the nephrons, the basic structural and functional units of the kidney (Figure 1). Nephron components filter the blood free of cells and large proteins, producing an ultrafiltrate composed of the other smaller circulating elements. The ultrafiltrate enters tubule segments to produce the final urine by removing (reabsorption) or adding (secretion) substances from or to the tubular fluid (Gueutin et al., 2012; Mount, 2014). Indeed, by adapting the quality composition of urine to the needs of the body, kidneys keep the organism in balance of water, hydrogen ion concentration, electrolytes, and minerals, and eliminate the toxic substances produced in the body. Deregulation of kidney functions may lead to severe pathological conditions affecting different tissues and organs.

Kidney diseases are worldwide the 12th leading cause of death and the 17th cause for loss of healthy life years (Glassock et al., 2017). They are classified into two major groups of pathologies depending on the length of the disease encompassing acute kidney injury (AKI), which is an abrupt reduction of kidney functions within 48 hours (Mehta et al., 2007; Rewa and Bagshaw, 2014), and chronic kidney disease (CKD) that is a gradual loss of renal function over years (Jha et al., 2013; Hill et al., 2016). AKI is associated with an high mortality rate (30–70%) and can have long-term consequences predisposing to CKD development (Coca et al., 2009). Due to the lack of adequate specific treatments, many patients (>2 millions worldwide) progress from CKD to end-stage renal disease and organ failure, requiring dialysis, or kidney transplantation (Hu and Coresh, 2017). Management of AKI and CKD represents a massive burden for the health care systems (Kerr et al., 2014), and CKD is on the rise due to the aging of the population and the clinical complications associated with diabetes and hypertension (Jobs et al., 2011; Tonelli and Riella, 2014). Therefore, there is an urgent need to increase our understanding on kidney disease pathogenesis to find new selective biomarkers or therapeutic candidates for drug development.

In this scenario, emerging evidence demonstrate the important role for lysosomal proteases cathepsins (Cts) in the onset and progression of kidney disease (Svara et al., 2010; Moallem et al., 2011; Ozkayar et al., 2015; Cocchiaro et al., 2016; Fox et al., 2016; Yamamoto-Nonaka et al., 2016; Conley et al., 2017). Lysosomes are ubiquitous organelles responsible for the catabolism and recycling of different types of macromolecules and constitute the major degradative compartment of the cell (Cuervo and Dice, 1998). They are involved in the renal epithelial molecular machinery underlying kidney physiology (Surendran et al., 2014). Two classes of proteins mediate lysosomal activity: integral lysosomal membrane proteins and soluble lysosomal hydrolases.

Among hydrolases, Cts are implicated in multiple cellular processes ranging from the processing of proteins and hormones to the regulation of cell cycle, autophagy, cell death, and immune response (Ciechanover, 2012). Altered expression and/or activity of Cts have been associated with a variety of human diseases (Reiser et al., 2010; Pišlar and Kos, 2014; Stoka et al., 2016). Since a growing number of studies deals with the involvement of Cts in kidney physiopathology, this review aims to highlight the most recent advances in our understanding of the molecular mechanisms by which lysosomal Cts promote kidney disease.

## Proteases cathepsins

To date more than 20 types of Cts have been identified in animals, plants, and microorganisms. In humans, 15 types of Cts have been reported, which can be classified into 3 distinct groups based on the amino acid that comprises the active site residue: serine (Cts A and G), cysteine (Cts B, C, H, F, L, K, O, S, V, X, W), and aspartate proteases (Cts D and E) (Table 1).

**Table 1:** Classification, tissue localization and disease involvement of human cathepsins.

**Cat**.	**Protease family**	**Aminoacids**	**Localization**	**Disease involvement**
A	Ser	480	Brain, skin, placenta, liver, kidney, platelets	Mucopolysaccharidosis (Pereira et al., 2016) Sialidosis (d'Azzo et al., 2015) Cardiomyopathies (Hua and Nair, 2015)
B	Cys	339	Liver, kidney, spleen, thyroid	Alzheimer's disease. (Schechter and Ziv, 2011) Atheriosclerosis (Hua and Nair, 2015) Cancer and metastasis (Gocheva and Joyce, 2007) Inflammatory lung disease (Zhang et al., 2015) Neurodegenerative disorders (Stoka et al., 2016) Rheumatoid arthritis and osteoarthritis (Pozgan et al., 2010) Kidney disease (Senatorski et al., 1998; Tao et al., 2005; Svara et al., 2010; Peres et al., 2013; Liu et al., 2015; Musante et al., 2015; Fox et al., 2016; Lim et al., 2016; Scarpioni et al., 2016; Wang et al., 2016; Conley et al., 2017)
C	Cys	463	Liver, lung, kidney, spleen, gut, placenta, T lymphocytes	Papillon-Lefèvre and Haim-Munk syndromes (Rai et al., 2010) Diabetes (Korpos et al., 2013) Inflammatory lung disease (Hamon et al., 2016) Neurodegenerative disorders (Stoka et al., 2016) Squamous tumors (Ruffell et al., 2013)
D	Asp	412	Spleen, kidney, liver, platelets	Atheriosclerosis (Hua and Nair, 2015) Cancer (Benes et al., 2008) Neurodegenerative disorders (Stoka et al., 2016) Neuronal ceroid lipofuscinosis (Benes et al., 2008) Obesity (Hua and Nair, 2015) Kidney disease (Moallem et al., 2011; Ozkayar et al., 2015; Cocchiaro et al., 2016; Fox et al., 2016; Yamamoto-Nonaka et al., 2016)
E	Asp	401	Brain, gut, skin, spleen, lung, kidney, lymph nodes, erythrocytes, adipocytes	Alzheimer's disease (Mackay et al., 1997) Cancer (Abd-Elgaliel et al., 2013; Kawakubo et al., 2014) Rosai-Dorfman disease (Paulli et al., 1994)
F	Cys	484	Brain, heart, skeletal muscle, testis, ovary, kidney, macrophages	Cancer (Vazquez-Ortiz et al., 2005; Ji et al., 2017) Kufs-disease (Peters et al., 2015) Neurodegenerative disorders (Stoka et al., 2016)
G	Ser	225	Skin, kidney, monocytes, neutrophils	Atheriosclerosis (Rafatian et al., 2013) Cardiovascular and cerebrovascular diseases (Herrmann et al., 2001) Chronic obstructive pulmonary disease (COPD), Crohn's disease, rheumatoid arthritis, cystic fibrosis (Kosikowska and Lesner, 2013) Papillon-Lefevre syndrome (Korkmaz et al., 2010) Glomerulonephritis and renal failure (Johnson et al., 1988; Sanders et al., 2004; Shimoda et al., 2007; Cohen-Mazor et al., 2014)
H	Cys	335	Liver, kidney, spleen	Cancer and metastasis (Gocheva and Joyce, 2007) Inflammatory lung disease (Bunatova et al., 2009) Rheumatoid arthritis (Jørgensen et al., 2011)
K	Cys	329	Lung, osteoclasts, macrophages, embryonic epithelial gastrointestinal cells, respiratory and urinary tracts	Atherosclerosis and obesity (Lafarge et al., 2010) Cardiac hypertrophy (Hua and Nair, 2015) Cancer (Husmann et al., 2008) Inflammatory lung disease (van den Brûle et al., 2005) Osteoarthritis (Saftig et al., 1998) Rheumatoid arthritis (Hao et al., 2015)
L	Cys	333	Liver, thyroid, kidney, macrophages	Alzheimer's disease (Schechter and Ziv, 2011) Neurodegenerative disorders (Stoka et al., 2016) Atherosclerosis and obesity (Lafarge et al., 2010; reviewed in Hua and Nair, 2015) Diabetes (Huang et al., 2003) Cancer and metastasis (Sudhan and Siemann, 2015) Rheumatoid arthritis and osteoarthritis (Solau-Gervais et al., 2007) Kidney disease (Cohen and Kretzler, 2003; Goulet et al., 2004; Reiser et al., 2004; Sever et al., 2007; Bauer et al., 2011; Haase et al., 2014; Sołtysiak et al., 2014; Carlsson et al., 2015; Liu et al., 2015; Garsen et al., 2016; Cao et al., 2017)
O	Cys	321	Liver, kidney, ovary, placenta	Breast cancer (Cairns et al., 2017)
S	Cys	331	Spleen, lymph nodes, heart	Alzheimer's disease (Schechter and Ziv, 2011) Atherosclerosis and obesity (Jormsjö et al., 2002; Lafarge et al., 2010; Hua and Nair, 2015) Diabetes (Jobs et al., 2013; Korpos et al., 2013) Cancer and metastasis (Gocheva and Joyce, 2007) Inflammatory lung disease (Bunatova et al., 2009) Rheumatoid arthritis and osteoarthritis (Pozgan et al., 2010) Kidney disease (Luhe et al., 2003; Aikawa et al., 2009; Carlsson et al., 2015; Figueiredo et al., 2015; Steubl et al., 2017)
V	Cys	334	Cornea, thymus, testis, liver, heart, kidney, colon, T lymphocytes	Atheriosclerosis (Yasuda et al., 2004) Cardiovascular disorders (Keegan et al., 2012; Leng et al., 2017) Neurological diseases (Funkelstein et al., 2012) Pulmonary sarcoidosis (Naumnik et al., 2015) Systemic sclerosis (Noda et al., 2013)
W	Cys	376	Spleen, lymph nodes, liver, heart, kidney	Leukemia (Kothapalli et al., 2003) Diabetes (Korpos et al., 2013) Gastroesophageal reflux disease (Raab et al., 2011) Inflammatory bowel disease or autoimmune gastritis (Buhling et al., 2002)
X	Cys	303	Liver, kidney, placenta, lung, heart, colon	Neuroinflammation and multiple sclerosis (Stoka et al., 2016; Allan et al., 2017) Cancer and metastasis (Nägler et al., 2004; Krueger et al., 2005; Wang et al., 2011)

Mainly localized in the lysosomes where their activity is facilitated by the lysosomal acidic environment, under certain circumstances, Cts can also be found in the intracellular and extracellular spaces (Stoka et al., 2001, 2005, 2016; Jordans et al., 2009). Indeed, leakage of CtD from the lysosome into the cytosol induces apoptosis (Liaudet-Coopman et al., 2006). In addition, Cts B, D, G, K, L, S, and X participate in the degradation of the major extracellular matrix components in various pathophysiological processes (Brix et al., 2008).

Almost all types of Cts share a common synthetic pathway (Ishidoh and Kominami, 2002). They are synthesized as inactive preproenzyme, and following translocation into the endoplasmic reticulum (ER), the N-terminal signal peptide of the precursor protein is cleaved with simultaneous N-linked glycosylation of the proenzyme (zymogen) (Erickson, 1989; Wiederanders et al., 2003). The propeptide is transported to the Golgi apparatus where it is further glycosylated and phosphorylated to form a mannose-6-phosphate protein that is recognized by the mannose-6-phosphate receptor and carried toward the lysosome where it is hydrolyzed to the active form. This general mechanism of Ct biosynthesis and transport may vary in some cases. The proteolytic cleavage of the zymogen may occur either through an autocatalytic process which is facilitated by the binding of the zymogen to glycosaminoglycans (GAGs) or through the action of other proteases (Dahl et al., 2001; Vasiljeva et al., 2005; Caglic et al., 2007).

**Figure 2:**
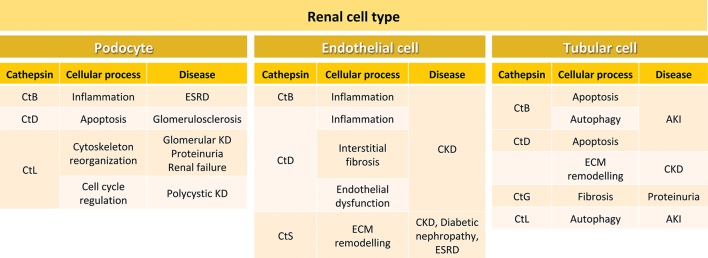
Cellular processes and kidney diseases involving cathepsins in different renal cell types. In podocytes, CtB participates in inflammation during ESRD, CtD is involved in apoptosis in glomerulosclerosis, and CtL plays a role in cytoskeleton reorganization and cell cycle regulation during glomerular kidney disease, proteinuria, renal failure and polycystic kidney disease. In endothelial cells, CtB and CtD are involved in inflammation. In addition, CtD participates in interstitial fibrosis and endothelial dysfunction during CKD. CtS is important in CKD, diabetic nephropathy and ESRD. In tubular cells, CtsB is involved in apoptosis and autophagy during AKI, and CtD in apoptosis and ECM remodeling during CKD. CtG participates in fibrosis during proteinuria, and CtL in autophagy in AKI. ESRD, end-stage renal disease; KD, kidney disease; CKD, chronic kidney disease; ECM, extracellular matrix; AKI, acute kidney injury.

Although Cts show similarities in their cellular localization and biosynthesis, they are expressed at different levels in tissues and organs (Table 1). While some Cts such as B, H, L, C, and O are ubiquitously expressed, other Cts such as F, K, S, V, X, and W show a more limited cell and tissue distribution and expression. The differences in tissue localization and expression levels suggest specific cellular functions for different Cts (Brix et al., 2008; Reiser et al., 2010; Stoka et al., 2016). The relevance of the Cts physiological roles in different organs and tissues is supported by multiple evidence demonstrating that abnormal levels or activity of Cts correlate with numerous human diseases, including inflammatory and cardiovascular diseases, neurodegenerative disorders, diabetes, obesity, cancer, kidney dysfunction, and others (Table 1). In particular, depending on the cell type localization, Cts B, D, L, and S regulate in the kidney different physiopathological processes, by activating signaling pathways that ultimately may result in kidney disease (Figure 2).

## Cathepsins in acute kidney injury (AKI)

AKI is characterized by a relatively sudden reduction, within 48 hours, in kidney function or production, processing, and excretion of ultrafiltrate by the kidney (decreased glomerular filtration rate, GFR) (Mehta et al., 2007). Permanent damage to the microvasculature with subsequent abnormalities in kidney structure and function are caused by AKI. Incomplete recovery from AKI leads to the development of CKD (Venkatachalam et al., 2015; Sud et al., 2016). To date, no effective treatments for AKI are available.

A variety of insults may promote the onset of AKI, leading all of them to epithelial tubular cell death. Increasing evidence demonstrates that ER dysfunction and mitochondrial stress causing tubular damage are important factors in the pathogenesis of AKI (Tábara et al., 2014; Ishimoto and Inagi, 2016; Duann and Lin, 2017; Galvan et al., 2017). Cts play important roles in the signaling pathways driving apoptotic and necrotic cell death, by degrading different substrates and/or contributing to mitochondrial destabilization (Turk et al., 2002; Stoka et al., 2005; Turk and Stoka, 2007). Increased expression levels and activation of CtB have been observed in the human proximal tubular epithelial cell line HK-2 undergoing to apoptosis (Wang et al., 2008). Autophagy induction in proximal tubular cells occurs during AKI (Livingston and Dong, 2014). The activity of CtB and CtL decreases when autophagy-lysosome pathway in HK-2 is disrupted by advanced glycation end products in diabetic nephropathy (Liu et al., 2015). Decreased activity of CtB correlates with an impairment of the autophagic flux and worsening of the renal function in a tubular epithelial cell model (Herzog et al., 2012). In a rat model of AKI, a significant decrease of CtB was detected in the affected proximal tubules, which correlated with increased severity of the histopathological lesions of the tubules (Svara et al., 2010). However, although autophagy causes cell death under certain conditions, a renoprotective role for autophagy in AKI has been established (Jiang et al., 2012). Urinary CtB levels have shown a strong inverse correlation with surrogate markers of nephron number in intrauterine growth-restricted neonates and pre-term infants, suggesting that urinary CtB activity may represent an useful tool for early predicting renal susceptibility to damage in low birth weight neonates (Aisa et al., 2016). Serum CtB concentration directly correlates with the loss of renal function in healthy individuals and the aging-related decrease of kidney function in the normal population (Wang et al., 2016).

The protease CtD is highly expressed in damaged tubular cells suggesting a possible contribution of CtD to cell death in AKI (Cocchiaro et al., 2016). During apoptosis, lysosomal membrane permeabilization allows translocation of CtD from the lysosome into the cytosol where it can exert its pro-apoptotic function. Cytosolic CtD cleaves Bid protein into tBid triggering the insertion of Bax protein into the mitochondrial membrane. This leads to cytochrome c release from the mitochondria into the cytosol, and the activation of pro-caspases 9 and 3 (Stoka et al., 2001). Enhanced CtD expression has been found in murine models of AKI (Kimura et al., 2012; Cocchiaro et al., 2016). CtD has been recently identified as a possible novel prognostic marker for AKI, as it is differentially regulated in urine from late/non recovered vs. early/recovered AKI patients (Aregger et al., 2014).

Translocation of CtL from the lysosome into the cytoplasm is a key event in the induction of glomerular kidney disease (Sever et al., 2007). The onset of proteinuria in kidney dysfunctions reflects a migratory event in the foot processes of the podocytes that correlates with the activation of CtL (Reiser et al., 2004; Cao et al., 2017). Three substrates have been described for cytosolic CtL in podocytes: CD2-associated protein, synaptopodin and dynamin (Sever et al., 2007; Mundel and Reiser, 2010; Yaddanapudi et al., 2011). These proteins are crucial for maintaining the normal cytoskeleton architecture of podocytes, and their degradation by CtL results in the reorganization of the actin cytoskeleton, proteinuria and renal failure (Reiser et al., 2010; Garsen et al., 2016). An emerging role of nuclear CtL in polycystic kidney disease comes out from the evidence that a CtL isoform lacking of a signal peptide localizes to the nucleus in S phase and processes the CDP/Cux transcription factor, thus regulating cell cycle progression (Goulet et al., 2004). The quantification of CtL has been demonstrated to provide a better predictive value for AKI than creatinine, urea and urine output (Haase et al., 2014). Genome expression studies performed with RNA from kidneys of 7-week-old male and female double transgenic rats (dTGRs), harboring human renin and angiotensinogen genes, showed that CtL was differentially expressed between the sexes and was strongly associated with the degree of renal injury (Bauer et al., 2011).

Finally, CtG has been identified as a critical component sustaining neutrophil-mediated acute tissue pathology and subsequent fibrosis after renal ischemia/reperfusion injury (Shimoda et al., 2007). It has been shown that CtG mediates marked changes in glomerular permeability *in vivo*, contributing to proteinuria (Johnson et al., 1988).

## Cathepsins in chronic kidney disease (CKD)

In spite of advance in the development of treatment approaches to improve outcomes, CKD is still associated with a high morbidity and mortality rate for patients affected by kidney dysfunctions (Hill et al., 2016; Glassock et al., 2017). AKI can contribute or worse the progression of CKD because of an abnormal or incomplete repair response (Chawla et al., 2014). The primary glomerular injury leads to a decreased post-glomerular flow, which finally results into peri-tubular capillary loss. Alternatively, renal injury can trigger an inflammatory response that recruits profibrotic cytokines such as transforming growth factor-β, and further induces the transformation of renal epithelial and endothelial cells to myofibroblasts (De Chiara and Crean, 2016; Cruz-Solbes and Youker, 2017). The histopathological hallmark of CKD is tubulointerstitial fibrosis, which is currently thought to be the best predictor to assess progression toward end-stage renal disease (Liu, 2006).

During CKD, CtD plays critical roles in inflammation and endothelial dysfunction (Erdmann et al., 2008; Ozkayar et al., 2015; Fox et al., 2016). Elevated expression levels of CtD have been found in human and murine damaged kidneys. Inhibition of CtD by Pepstatin A in murine models of progressive CKD resulted in a reduction of interstitial fibrosis (Fox et al., 2016). CtD inhibition led to an increase in extracellular protease activity of urokinase-type plasminogen activator (uPA) due to altered lysosomal recycling; UPA processes plasminogen into plasmin, which can degrade extracellular matrix proteins (Eddy, 2009). A role for CtD in podocytes, responsible for maintaining the ultrafiltration barrier thus preventing urinary protein loss, has also been reported (Yamamoto-Nonaka et al., 2016). In a podocyte-specific knock-out mouse model, the absence of CtD resulted in podocyte apoptotic cell death, and in age–dependent, late–onset glomerulosclerosis (Alghamdi et al., 2017). Therefore, CtD activity in kidney could be different depending on the cell type, and further studies will be required to clarify this issue. In CKD, CtD serum levels were significantly higher and correlated with endothelial dysfunction in patients (Ozkayar et al., 2015). However, no correlation was found between serum CtD levels and traditional cardiovascular risk factors, indicating that enhanced CtD could be a selective risk factor for endothelial dysfunction in kidney disease.

Altered levels of CtB activity have been detected under pathological processes in kidney (Ling et al., 1998; Senatorski et al., 1998; Svara et al., 2010). Toll-like receptor 3 (TLR3), which activates both the innate and adaptive immune systems, is cleaved and activated by CtB (Garcia-Cattaneo et al., 2012). CtB-dependent activation of TLR3 leads to the activation of the transcription factors NF-κB and interferon regulatory factor 3, resulting into the production of type I interferons and pro-inflammatory cytokines such as IL-6 and IL-8 (Kawasaki and Kawai, 2014). In the kidney, inflammation promotes the progression of glomerular sclerotic pathologies resulting in end-stage renal disease (Anders and Muruve, 2011; Lim et al., 2016). It has been demonstrated that CtB mediates the signaling pathway activating the inflammasome, a large multiprotein complex containing NOD-like receptor with pyrin domain 3 (NLRP3) which triggers the production of proinflammatory cytokines in response to infection and tissue injury (Conley et al., 2017). NLRP3 inflammasome activation by CtB may promote glomerular inflammation and other cell damages resulting into glomerular injury and end-stage renal disease. Inflammasome activation may occur not only in immune cells but also in residential cells such as endothelial cells and podocytes in the glomeruli (Conley et al., 2017). Thus, NLRP3 inflammasome has been suggested as a potential target for the treatment of progressive CKD (Scarpioni et al., 2016). A correlation between serum CtB concentration and the age-related decline in renal function has been described in healthy individuals (Wang et al., 2016). CtB has also been shown to be involved in diabetic nephropathy (Musante et al., 2015). Other reports demonstrate a reduction of CtB activity during polycystic kidney disease (Schaefer et al., 1996; Hartz and Wilson, 1997; Tao et al., 2005), puromycin induced nephrosis (Huang et al., 1999), and rat and human diabetic nephropathy (Shechter et al., 1994; Grzebyk et al., 2013; Peres et al., 2013). Conversely, CtB expression increased in unilateral ureteric obstruction mouse model, however, its inhibition led to no reduction in kidney fibrosis (Fox et al., 2016).

The expression of CtL results to be enhanced in various glomerular diseases such as focal segmental glomerulosclerosis, membranous glomerulonephritis, and diabetic nephropathy (Baricos et al., 1991; Sever et al., 2007). Induction of CtL expression in podocytes has been associated with the development of proteinuria in puromycin aminonucleoside induced-kidney failure (Reiser et al., 2004), and streptozotocin-induced diabetic nephropathy (Garsen et al., 2016). CtL can contribute to the development of kidney disease by different mechanisms. Cytoplasmic CtL cleaves the GTPase dynamin resulting in podocyte failure and proteinuria (Sever et al., 2007). In addition, CtL activates proteins such as heparanase that are involved in the pathogenesis of diabetic nephropathy (Garsen et al., 2016). Interestingly, CtL expression levels resulted to be lower in males than in females, but the increase in CtL detected with disease progression was greater in males. This evidence strongly suggests that estrogens regulate CtL expression and activity (Bauer et al., 2011). In CKD patients, serum CtL activity is markedly elevated and its levels positively correlate with the severity of proteinuria (Cohen and Kretzler, 2003; Sever et al., 2007; Cao et al., 2017). The presence and severity of proteinuria in patients with CKD is associated with higher mortality and morbidity (Hemmelgarn et al., 2010; Garsen et al., 2016). Elevated CtL activity correlates with higher hospital admission rates in CKD patients (Cao et al., 2017). Urinary excretion of CtL was higher in children with type 1 diabetes mellitus with respect to healthy patients (Sołtysiak et al., 2014).

In contrast with other Ct members, CtS remains catalytically active under neutral pH (optimum pH values, 6.0–7.5) and its main physiological role is outside the lysosome. Intracellularly, CtS has an important role in the intrinsic apoptotic pathways inducing cleavage of both caspase-3 and poly ADP ribose polymerase (Wang et al., 2015). CtS can translocate to the cell surface and be secreted into the extracellular milieu, participating in the degradation of extracellular matrix proteins (Jordans et al., 2009; Wilkinson et al., 2015). Beside its ability to degrade fibers, CtS may activate the protease-activated receptor-2 (PAR2) in endothelial cells (Elmariah et al., 2014). Indeed, *in vitro* studies demonstrated that CtS may damage the integrity and barrier function of glomerular endothelial cells (Aikawa et al., 2009; Lafarge et al., 2010). In human and mouse type 2 diabetic nephropathy, CtS mRNA resulted to be expressed only in CD68(+) intrarenal monocytes, while the protein was found along endothelial cells and inside proximal tubular epithelial cells (Kumar et al., 2016). High circulating levels of CtS have been correlated with increased mortality risk in the human population (Jobs et al., 2011) because of its involvement in the complex pathways leading to cardiovascular disease, cancer and impaired kidney function (Feldreich et al., 2016). *In vivo* studies demonstrated that CtS-induced elastolysis stimulates arterial and aortic valve calcification in CKD, suggesting that CtS might be a therapeutic target to prevent cardiovascular complications in CKD (Aikawa et al., 2009). Up-regulation of CtS has been detected in ochratoxin A-induced nephropathy (Luhe et al., 2003). Furthermore, selective CtS inhibition attenuates atherogenesis in hypercholesterolemic mice with CKD (Figueiredo et al., 2015). In mice, serum levels of CtS and markers of inflammation-related endothelial dysfunction, such as soluble tumor-necrosis-factor receptors (sTNFR) 1 and 2, increase with the decline of estimated GFR, while in human cohortes an increase of GFR was associated with a decrease of CtS (Steubl et al., 2017). However, in patients with end-stage renal disease, high levels of CtS were associated with sTNFR1/2 activation (Carlsson et al., 2015). These findings indicate that CtS activity increases with CKD progression, thus representing a potential marker of disease progression.

## Conclusions and perspective

Kidney disease, characterized by the progressive loss of kidney functions, occurs through different steps of damage leading to organ failure and end-stage renal disease. Due to the lack of specific treatments to stop disease progression (Mehta et al., 2007; Black et al., 2010), kidney disease remains an important clinical problem affecting millions of people worldwide (Jha et al., 2013; Hu and Coresh, 2017). In addition, the traditional clinical markers used to assess and monitor kidney function such as serum creatinine, GFR, and the presence of proteinuria often miss the early stages of the disease delaying essential treatment (Mårtensson et al., 2012; Haase et al., 2014; Wasung et al., 2015). Indeed, both of the two major groups of kidney disease, AKI and CKD, are still associated with increasing morbidity and mortality (Coca et al., 2009; Kerr et al., 2014; Hill et al., 2016; Glassock et al., 2017). Therefore, there is an urgent need to better understand the biological events driving AKI and CKD in order to either find more accurate and sensitive biomarkers of cell injury that may predict disease progression or identify critical cellular and molecular mediators that may provide novel therapeutic targets. Lysosomal Cts have emerged in the recent years as important players in kidney disease, thus suggesting their detection as early diagnostic approach. Moreover, targeting Cts or their downstream signaling seems a promising treatment strategy to slow down kidney disease progression. Nevertheless, further studies are required to assess the suitability, specificity and drugability of Cts in human kidney disease.

## Author contributions

PC, VDP, LMP, and AM has conceived, designed the work, written and revised the manuscript. RDM, ST, LA, and AP have collaborated to design, to write and revise the manuscript.

### Conflict of interest statement

The authors declare that the research was conducted in the absence of any commercial or financial relationships that could be construed as a potential conflict of interest.
